# Aleocharine rove beetles (Coleoptera, Staphylinidae) associated with *Leptogenys* Roger, 1861 (Hymenoptera, Formicidae) II. Two new genera and two new species associated with *L. borneensis* Wheeler, 1919

**DOI:** 10.3897/zookeys.59.511

**Published:** 2010-10-01

**Authors:** Munetoshi Maruyama, Christoph von Beeren, Volker Witte

**Affiliations:** 1 The Kyushu University Museum, Fukuoka, 812-8581 Japan; 2 Department Biologie II, Ludwig-Maximilians-Universität München, Großhaderner Straße 2, 82152 Planegg-Martinsried, Germany

**Keywords:** Myrmecophily, *Parawroughtonilla* gen. n., *Leptogenonia* gen. n., *Witteia* gen. n., *Wroughtonilla* genus group, new species, Malaysia

## Abstract

Two new genera and two new species of Aleocharinae (Staphylinidae) from Malaysia are described: Parawroughtonilla Maruyama, **gen. n.** (type species: Parawroughtonilla hirsutaMaruyama, **sp. n.**), Leptogenonia Maruyama, **gen. n.** (type species: Leptogenonia roslii Maruyama, **sp. n.**), which are associated with Leptogenys borneensis Wheeler, 1919. They are closely related and share a unique character state of the aedeagus.

## Introduction

Recently, the junior authors (CvB and VW) collected two species of rove beetles from colonies of Leptogenys borneensis Wheeler, 1919 that apparently belong to different genera of the tribe Lomechusini (subfamily Aleocharinae). Kistner et al.(2008) identified them as “Maschwitzia ulrichi” and “Neowroughtonilla steghausae” that are known to be associated with colonies of Leptogenys distinguenda (Emery, 1887) and Leptogenys diminuta (F. Smith, 1857) respectively (Kistner 1989), and they recorded both for the first time with Leptogenys borneensis. However, the Leptogenys-associated rove beetles generally have strict host specificity, i.e., one rove beetle species is associated with only one or two closely related host ant species (Maruyama, unpublished data; von Beeren and Witte, personal observations). The first author found that the identifications of Kistner et al. (2008) are not correct, and both species can not conclusively be assigned to any known aleocharine genera and species.

This paper, the second part of the series on aleocharine rove beetles associated with Leptogenys, describes two new genera and two new species associated with Leptogenys borneensis, including discussion of their systematic positions.

## Materials and methods

**Figures 1–2.**
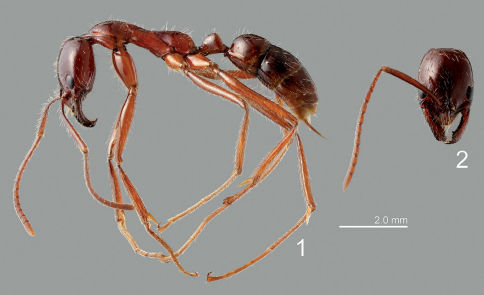
Leptogenys borneensis. **1** lateral view **2** head.

Between August 2007 and September 2009 a total of 11 months of field work was performed in a regenerated, secondary dipterocarp lowland rainforest at the Field Studies Centre of the University of Malaya (Kuala Lumpur), which is located in Ulu Gombak, Malaysia (03°19.4796N; 101°45.1630E, altitude 230 m). We located Leptogenys borneensis (Figs 1–2) nest sites by back-tracking ant raiding trails. Accordingly, we marked the nest sites and checked every 30 min for an approaching ant migration between 8 p.m. and 3 a.m. Since both rove beetle species take part in the ants’ migrations, they can be detected and collected with the help of aspirators during these activities. Their behavior was observed in the laboratory in ant nest fragments (for further information on methods see Witte et al. 2008). After behavioral analysis, the specimens were stored in 90% Ethanol.

Morphological analyses were performed as in the first part of this series (Maruyama et al. 2010). Specimens are deposited in the senior author’s collection in the Kyushu University Museum (KUM). Measurements are given in millimeters and are abbreviated as follows: antennal length (AL); body length (BL); fore body length, from front margin of head to apices of elytra (FBL); hind tibial length (HTL); head length (HL); head width (HW); pronotal length (PL); pronotal width (PW).

## Taxonomy

### Parawroughtonilla

Maruyama gen. n.

urn:lsid:zoobank.org:act:3EB3DA5D-454F-434B-90FB-ECAB682BBFBB

Figs 3 6 14 

#### Type species.

Parawroughtonilla hirsuta Maruyama, sp. n.

#### Etymology.

A combination of the Greek *para*-, meaning near, and Wroughtonilla Wasmann, 1899, a closely related genus. Gender, feminine.

#### Diagnosis.

This genus is rather similar to Togpelenys Kistner, 1989 in body shape and punctation of body surface, but may easily be distinguished from it by the smaller eyes, and the abdomen being densely covered with setae.

#### Description.

##### Body

**Figures 3–5.**
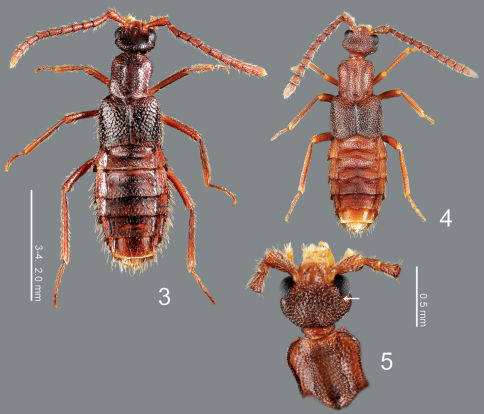
**3** Parawroughtonilla hirsuta gen. et sp. n., dorsal habitus **4** Leptogenonia roslii gen. et sp. n., dorsal habitus **5** ditto, head and pronotum, dorsal view

(Fig. 3) elongate, slightly flattened; surface of fore body strongly rugose, shining.

**Figures 6–9.**
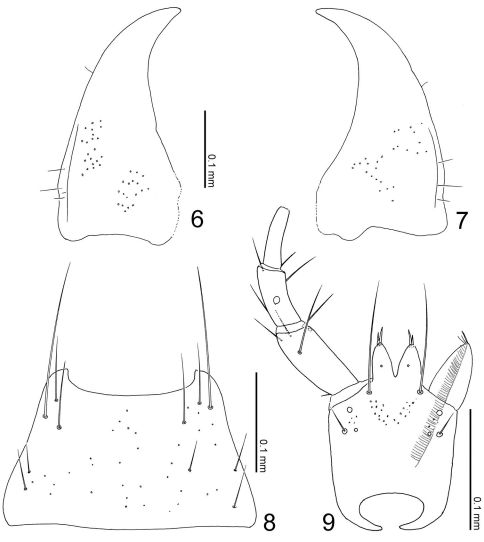
Mouthparts of Parawroughtonilla hirsuta gen. et sp. n. **6** left mandible, dorsal view (prostheca not shown) **7** right mandible, dorsal view (ditto) **8** mentum, ventral view **9** labium, ventral view.

**Figures 10–14.**
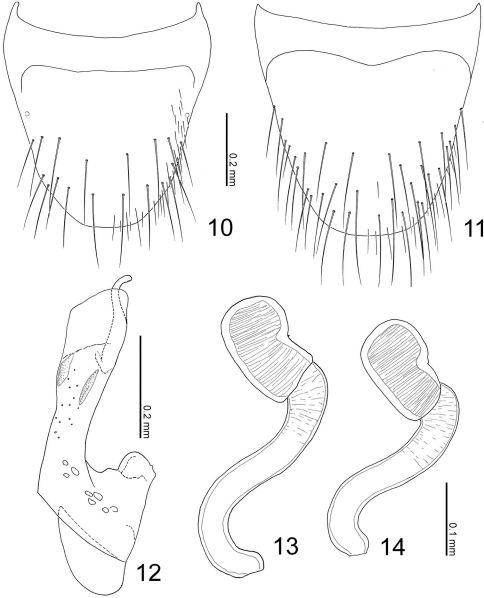
Terminalia of Parawroughtonilla hirsuta gen. et sp. n. **10** Male tergite VIII, dorsal view **11** male sternite VIII, ventral view **12** median lobe of aedeagus, lateral view **13, 14** spermathecae.

##### Head

(Fig. 3) transverse, depressed above, widest at eyes that are large, less than 1/2 as long as head; clypeus truncate apically. Labrum weakly emarginate antero-medially, with 2 setae and 4 long setulae along anterior margin, with sparse pseudopores around lateral areas. Mandibles (Figs 6, 7) almost symmetric, gently curved, each apex acutely pointed; inner margin of left mandible (Fig. 6) with a small notch. Mentum (Fig. 8) trapezoidal, with several thick setae, with sparse pseudopores. Labium (Fig. 9) broad; prementum with a setal pore, in which seta is rather long, and 2 real pores in its inner side, with several pseudopores around real pores and base of medial seta; apodeme without median projection, with lateral projection short, curved apically; ligula long, each lobe with 2 large setula; labial palpus with segment I long and apically dilated; segment II 2/3 as long as I; segment III thin, narrowed apically, slightly shorter than II.

##### Pronotum

(Fig. 3) slightly convex, with a broad longitudinal groove medially; sides weakly margined, i.e., superior marginal line somewhat obscured by rugose punctures continuing from disc. Mesocoxal cavity well margined; process of metaventrite narrow, pointed at apex.

##### Elytra

(Fig. 3) apically widened, laterally with a pair of carinae that are not clear, with large rugose punctures.

##### Legs

(Fig. 3) very long, thin; femora slightly narrowed apically; tibiae somewhat widened from around middle to basal 1/3, their bases constricted; tibiae somewhat thin.

##### Abdomen

(Fig. 3) rather expanded, widest around segment IV; surface densely punctured, shining. Median lobe of aedeagus (Fig. 12) with apical lobe covered by exposed inner sac which is fused with apical margin of aedeagus and well sclerotized. Paramere with apical lobe slightly widened apically, somewhat constricted around middle.

#### Parawroughtonilla hirsuta

Maruyama sp. n.

urn:lsid:zoobank.org:act:8C50277F-D6DA-4118-A57D-08B755272381

Figs 3 6 14 

##### Etymology.

In referring the hairy body.

##### Type series.

Holotype, male, Ulu Gombak (University Malaya Field Studies Centre, 03°19.479N; 101°45.170E, 230 m alt.), Selangor, Malaysia, VIII 2008, C. von Beeren, from the colony of Leptogenys borneensis (mouthparts and terminalia dissected and mounted in Euparal) (KUM). Paratypes: same data as holotype (1 female, 2 sex?); same data, but III 2008, C. von Beeren & V. Witte (3 males, 1 female).

##### Type locality.

Ulu Gombak, Selangor, Malaysia.

##### Distribution.

Peninsular Malaysia.

##### Symbiotic host.

Leptogenys borneensis.

##### Diagnosis.

This species is similar to Togpelenys gigantea in general appearance, but distinguished from it by the smaller body, the dense setation on the body surface and the presence of a superior marginal line of the pronotal hypomeron. This species is found together with Leptogenonia roslii in the same host colony, and can be easily distinguished from it by the larger body and the longer and denser setation on the body surface.

##### Description.

###### Body

(Fig. 3) color reddish brown, but head and elytra slightly darker. Head (Fig. 3) moderately covered with long erect setae; surface somewhat rugose. Antennae (Fig. 3) long; all segments longer than wide; segments III-X almost twice as long as wide; segment XI elongate. Pronotum (Fig. 3) longer than wide (width/length = 0.86), subparallel-sided, with anterior margin rounded, with posterolateral corners angled, produced laterally; surface moderately covered with long erect setae, which are poorly differentiated from macrosetae. Elytra (Fig. 3) moderately covered with long erect setae. Abdomen (Fig. 3) with sternites moderately covered with long erect setae, with tergite VIII (Fig. 10) rounded apically, with 9 macrosetae; sternite VIII (Fig. 11) rounded apically; tergite IX with 4 macrosetae; tergite X with 5 macrosetae postero-laterally.

###### Male:

sternite VIII (Fig. 11) with around 18 macrosetae. Median lobe of aedeagus (Fig. 12) with short parameral crest; apical lobe curved near apex.

###### Female:

sternite VIII with 14–16 macrosetae. Spermatheca (Figs 13–14) with basal part slightly dilated apically, twice curved near base and apex; apical part short.

BL, ≈ 4.1–4.5; FBL, ≈ 2.1–2.3; HL, 0.606–0.623; HW, 0.715–0.740; AL, ≈ 2.4–2.6; PL, 0.825–0.881; PW, 0.708–0.756; HTL, 1.270–1.350.

#### Leptogenonia

Maruyama gen. n.

urn:lsid:zoobank.org:act:BBAB7FB4-0580-4D85-85C7-FF95F0731D4F

##### Type species.

Leptogenonia roslii Maruyama, sp. n.

##### Etymology.

A combination of the host ant genus name Leptogenys and “-nia” that is the end of a lomechusine genus name Myrmedonia Erichson, 1837, in the same manner as Aenictonia Wasmann, 1900 which is associated with Aenictus ants.

##### Diagnosis.

This genus is similar to Maschwitzia Kistner, 1989 in body shape and punctation of body surface, but may easily be distinguished from it by the smaller body, the head with post-ocular ridges (Fig. 5: arrow), the temples being convex and the shorter legs. The head capsule structure is similar to those of Aenictonia and Anommatochara Wasmann, 1915 but is distinguished from those genera by the elytra without a pair of medial carinae.

##### Description.

###### Body

(Fig. 4) elongate, flattened; surface of fore body rugose, matte.

###### Head

**Figures 15–18.**
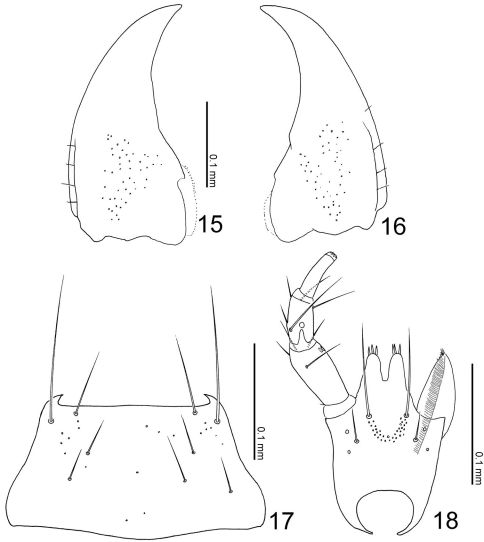
Mouthparts of Leptogenophila roslii gen. et sp. n. **15** left mandible, dorsal view (prostheca not shown) **16** right mandible, dorsal view (ditto) **17** mentum, ventral view **18** labium, ventral view.

(Figs 4, 5) transverse, depressed above, with post-ocular ridges (Fig. 5: arrow), widest at temples that are quite convex; occiput convex, with a pair of small tubercules; eyes large, less than 1/4 as long as head; clypeus truncate apically. Labrum weakly emarginate antero-medially, with 3 setae and 3 long setulae along anterior margin, sparsely with pseudopores around lateral areas. Mandibles (Figs 15–16) almost symmetric, gently curved, each apex acutely pointed; inner margin of right mandible (Fig. 16) with a small notch. Mentum (Fig. 17) trapezoidal, with several thick setae, very sparsely with pseudopores. Labium (Fig. 18) broad; prementum with a setal pore, in which seta is very long, and 2 real pores in its outer side, with several pseudopores around base of medial seta; apodeme without median projection, with lateral projection short, curved apically; ligula long, each lobe with 3 large setula; labial palpus with segment I long and apically dilated; segment II 4/3 as long as I, with membranous notch; segment III thin, narrowed apically, slightly as long as II.

###### Pronotum

(Fig. 4) slightly convex, with a broad longitudinal groove medially, its lateral lines convex as a pair of carinae, sides well margined and elevated, depressed along margins. Mesocoxal cavity well margined; process of metaventrite narrow, rounded at apex.

###### Elytra

(Fig. 4) apically widened, laterally with a pair of carinae; postero-inner margin of elytron obliquely truncate.

###### Legs

(Fig. 4) moderate in length; femora slightly narrowed apically near apex; tibiae somewhat widened from around middle to basal 1/3, their bases constricted; tarsi somewhat thin.

###### Abdomen

(Fig. 4) slightly expanded, widest around segment IV, well convex above; surface densely punctured, matte; segments II-IV emarginated posteromedially; segments III and IV with a pair of large depressions around base. Median lobe of aedeagus (Fig. 22) with apical lobe covered by exposed inner sac which is fused with apical margin of aedeagus and well sclerotized. Paramere with apical lobe slightly widened apically.

**Figures 19–23.**
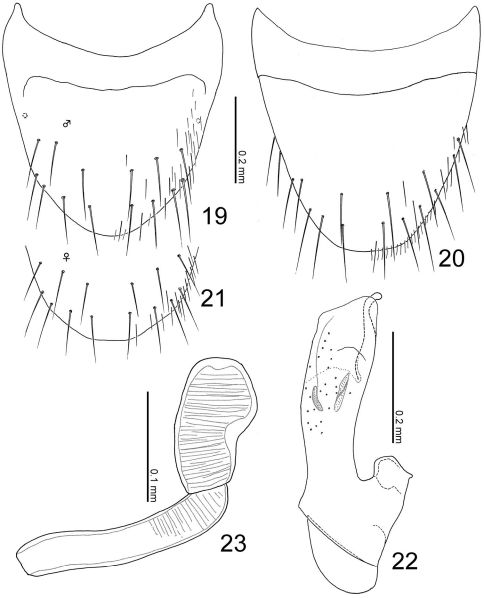
Terminalia of Leptogenophila roslii gen. et sp. n. **19** Male tergite VIII, dorsal view **20** male sternite VIII, ventral view **21** female tergite VIII, apical part, dorsal view **22** median lobe of aedeagus, lateral view **23** spermatheca.

#### Leptogenonia roslii

Maruyama sp. n.

urn:lsid:zoobank.org:act:24E09104-DF74-428E-8473-E0C0E4F211D6

Figs 4–5 15 23 

##### Etymology.

Dedicated to Dr. Rosli Hashim for his great support to field researchers in the Peninsular Malaysia that has resulted in numerous contributions to the knowledge on tropical animals.

##### Type series.

Holotype, male, Ulu Gombak (University Malaya Field Studies Centre, 03°19.479N; 101°45.170E, 220–250 m alt.), Selangor, Malaysia, III 2009, C. von Beeren & V. Witte, from the colony of Leptogenys borneensis (KUM). Paratypes: same data as holotype but VIII 2008, C. von Beeren (1 female, 1 sex?: KUM).

##### Type locality.

Ulu Gombak, Selangor, Malaysia.

##### Distribution.

Peninsular Malaysia.

##### Symbiotic host.

Leptogenys borneensis.

##### Diagnosis.

This species is similar to the members of the genus Maschwitzia but is easily distinguished from them by the smaller body and the shorter legs. This species is found together with Parawroughtonilla hirsuta in the same host colony, but it can be easily distinguished by the smaller body and the shorter and sparser setation on the body surface, especially by elytra lacking any setae.

##### Description.

###### Body

(Fig. 4) color pale reddish-brown, but head, pronotal lateral margins, elytra and abdominal segments V-VI darker. Head (Figs 4–5) moderately covered with short recumbent setae; surface somewhat rugose. Antennae (Fig. 3) long; all segments longer than wide; segments III-X almost 1.5 times as long as wide; segment XI conical. Pronotum (Fig. 4) longer than wide (width/length = 0.88–0.91), with anterior margin truncate, constricted around basal 1/3, with posterolateral corners rounded, produced laterally; surface without setae. Elytra (Fig. 4) with surface moderately covered with short recumbent setae. Abdomen (Fig. 4) with sternites moderately covered with long recumbent setae, with tergites moderately covered with very short recumbent setae; tergite VIII (Figs 19, 21) with 7 macrosetae; sternite VIII (Fig. 20) rounded apically, with 8 macrosetae; tergite IX with 4 macrosetae; tergite X with 4 macrosetae postero-laterally.

###### Male:

tergite VIII rounded apically. Median lobe of aedeagus (Fig. 22) with short parameral crest; apical lobe curved near apex.

###### Female:

tergite VIII slightly truncate apically. Spermatheca (Fig. 23) with basal part almost straight but slightly curved at apex; apical part large, 1/2 as long as basal part.

BL, ≈ 3.4–4.3; FBL, ≈ 1.8–2.0; HL, 0.538–0.555; HW, 0.625–0.644; AL, ≈ 2.0–2.1; PL, 0.725–0.748; PW, 0.644–0.656; HTL, 0.913–0.925.

## Behavioral observations

Both rove beetle species are highly integrated in the host ant society. They move undisturbed in between migrating ants, interact frequently with their host ants in laboratory nests and are overall treated peacefully. Both species fed on host diet (crickets) in laboratory nests, and they never preyed on any life stage of their host ants, suggesting a kleptoparasitic lifestyle (von Beeren et al. in press).

## Discussion

Kistner et al. (2008) recorded Maschwitzia ulrichi and Neowroughtonilla steghausae from Leptogenys borneensis. This report is based on the material collected by CvB and VW and represents misidentifications of these species. Leptogenys-associated rove beetles are highly host species specific as mentioned above. The authors CvB and VW have not collected any other species than Parawroughtonilla hirsuta and Leptogenonia roslii in their examinations of eightdifferent Leptogenys borneensis colonies after the report of Kistner et al. (2008). At least around the type locality Ulu Gombak, Malaysia, Parawroughtonilla hirsuta and Leptogenys roslii are the only myrmecophilous rove beetles found in Leptogenys borneensis colonies.

Parawroughtonilla and Leptogenonia both belong to the Wroughtonilla genus-group along with several other genera sharing several character states (Maruyama et al. 2010).

Parawroughtonilla hirsuta and Leptogenonia roslii are considerably different in their habitus, i.e., head, pronotum and abdominal structures. However, the states of the median lobe of aedeagus are almost the same, especially the apical lobe covered by the exposed inner sac which is completely fused with apical margin of median lobe and well sclerotized. This character state is apparently apomorphic and unique within the Wroughtonilla genus-group, i.e. it could be a synapomorphy for both species.

Leptogenonia is well characterized by the head structure: presence of the post-ocular ridges, the well convex temples and the occiput with a pair of tubercules. These character states are also observed in the genera Aenictonia and Anommatochara of the Wroughtonilla genus group that are mainly distributed in Africa (one species Aenictonia thailandica Seevers, 1965 is known from Thailand) which are associated with the army ant genera Aenictus Shuckard, 1840 and Dorylus Fabricius, 1793. However, the states of the aedeagus are very different between Leptogenonia, Aenictonia, and Anommatochara. Some Leptogenys species show army-ant life habits, comparable to the classic army ants of Ecitoninae, Aenictus and Dorylus (Kronauer 2009). Therefore similarities of the head structures evolved probably convergently between these aleocharines inhabiting colonies of army ants and ants that have army-ant life habits.

## Supplementary Material

XML Treatment for Parawroughtonilla
